# The role of surface and structural similarities in the retrieval of realistic perceptual events

**DOI:** 10.1111/bjop.12747

**Published:** 2024-11-13

**Authors:** Lucas Raynal, Evelyne Clément, Emmanuel Sander

**Affiliations:** ^1^ IDEA Lab, Faculty of Psychology and Educational Sciences, University of Geneva Genève Switzerland; ^2^ Paragraphe Lab, EA 349, CY Cergy Paris University and University Paris 8 Gennevilliers France

**Keywords:** analogy, ecological validity, memory retrieval, multimodality, perceptual richness, transfer

## Abstract

This study investigated whether structural similarities (i.e. abstract frames, e.g. *once bitten twice shy*) can prevail over surface similarities (i.e. contexts, e.g. *restaurant*) in driving the retrieval of realistic events involving dynamic, multimodal and perceptually crowded data. After watching an initial set of video clips, participants had to indicate whether a new video clip, that shared surface similarities with an initial event and structural similarities with another one, elicited a retrieval. The results of Experiment 1A showed that retrieval was more likely to be elicited by structural rather than by surface similarities. Experiment 1B confirmed that the surface similarities manipulated in this study were strong enough to elicit substantial retrievals when the competing structural match was neutralized. The pattern of results obtained in Experiment 1A remained unchanged when the number of unrelated video clips within the initial set was increased. The findings suggest that structurally based retrievals still prevail when familiar structures underlie realistic perceptual events. They open new perspectives regarding the settings that promote structurally based retrievals in educational contexts where unfamiliar principles are introduced.

## BACKGROUND

Analogical thinking is acknowledged to be a core cognitive mechanism, as it allows one to see past irrelevant contextual differences (i.e. *surface* or *superficial* level) and realize that two situations sharing a common abstract principle (i.e. *structure* level) are deeply similar (Gentner, 1983; Hofstadter & Sander, 2013; Holyoak & Thagard, 1995). This process is commonly divided into (at least) two subprocesses: the *retrieval* (also called reminding, access or recall) of a source situation from long‐term memory (LTM), and its *mapping* to the target cue situation once both representations are held in working memory (WM, Gentner & Maravilla, 2018; Goldwater & Schalk, 2016; Holyoak, 2012). While it is widely accepted that the systematic search for the relevant correspondences between two analogues during mapping generally leads to discovering their structural similarities (Markman & Gentner, 1993; Spellman & Holyoak, 1996), the type of similarities, or cues, driving retrieval is still a controversial issue in the current literature (Olguín et al., 2022; Raynal et al., 2020). Informing whether structural similarities can prevail over surface similarities in guiding retrieval is crucial, since it can be a precondition to spontaneously interpret incoming experiences through relevant analogies. In real‐life, however, structurally based retrievals require impressive abstraction capacities, since they require to extract structures from a dynamic flow of perceptual data received from different modalities (Chalmers et al., 1992). Thus, the experimental settings should approach or ideally reproduce this perceptual complexity to ensure the generalizability of the findings to how retrieval operates in real life. In the present contribution, we adopted a new experimental paradigm based on the presentation of video clips as a proxy for real‐life experiences to test the role of surface and structural similarities in the retrieval of realistic events.

### Surface versus structural similarities: which ones prevail in retrieval?

The superiority of surface over structural similarities has been supported by several studies showing that superficially similar disanalogues are more likely to be retrieved than superficially dissimilar analogues when both types of matches are confronted with each other in LTM (Gentner et al., 1993; Jamrozik & Gentner, 2020; Minervino & Trench, 2024; Trench et al., 2020;). The weak influence of structural similarities in retrieval was supported by studies demonstrating that participants have difficulty retrieving a superficially dissimilar case from memory when presented with another example of the same novel abstract principle (Gentner et al., 2009; Gick & Holyoak, 1980; Keane, 1987; Ross, 1987; Ross, 1989; Snoddy & Kurtz, 2021). The fact that superficially similar disanalogues are more likely to be retrieved than such superficially dissimilar analogues when both types of matches are confronted in LTM has suggested that surface similarities prevail in analogical retrieval. For instance, Jamrozik and Gentner (2020) found evidence that a target cue example (e.g. a description of a *positive feedback loop* between ice melt and the raise of temperature) more frequently elicited the retrieval of a superficially similar disanalogue (e.g. a description of an unrelated phenomenon from *environmental science*) than a superficially dissimilar analogue (e.g. a description of a *positive feedback loop* between a microphone and a speaker).

In line, Minervino & Trench, 2024, (Experiment 1) found that a target cue situation (e.g. a scientific attempt to reproduce quakes in an experimental zone monitored by a rescue firefighter in a helicopter) led to higher retrieval rates of a superficially similar disanalogue movie (e.g. *San Andreas*, in which a firefighter drives a helicopter to rescue his daughter during an apocalyptic earthquake) than a superficially dissimilar analogue movie (e.g. *Jurassic Park*, in which there is an attempt to reproduce dinosaurs to exhibit them in a public park). This body of evidence has led to the idea that surface similarities overcome structural similarities in analogical retrieval. However, an alternative explanation is that the paucity of structurally based retrieval in these studies is due to participants' lack of familiarity with the structure underlying the experimental stimuli, which prevents them from encoding structural cues (Dunbar, 2001; Hofstadter & Sander, 2013; Popov et al., 2017, Popov et al., 2020). In other words, analogical retrieval would not be guided by surface similarities per se, but only when the alternative structural similarities involve unfamiliar principles (e.g. *convergence schema*, *permutations*, *contingent contract*, *positive feedback loop, problem as a solution, reproduction of dangerous natural phenomena*, etc.).

The reliance on familiar structures (e.g. *sour grapes*, *making a deal to avoid a bad situation*, etc.) as opposed to surface features (e.g. *hunting context*, *pizza restaurant context*) as retrieval cues has been investigated more directly in story‐recall studies (Catrambone, 2002; Gentner et al., 1993; Raynal et al., 2020; Trench et al., 2020; Wharton et al., 1994, 1996). Using such a task, Gentner et al. (1993) presented participants with a set of source stories before introducing a set of target cue stories, some of which shared either surface or structural similarities with one of the source stories. Consistent with the surface superiority hypothesis, the main finding was that participants retrieved superficially similar disanalogues more frequently than superficially dissimilar analogues. However, Raynal et al. (2020) provided empirical evidence showing that superficially similar disanalogues from Gentner et al. (1993) share a residual structural overlap that may have inflated their retrieval rates. For instance, two superficially similar disanalogues involved a bird making a deal with a hunter to avoid being killed, and the scenarios differed only at the end of the stories, with the deal upheld in one story and a betrayal in the other. Raynal et al. (2020) devised a story‐recall study using a ‘competition’ paradigm (Wharton et al., 1994, 1996), in which each target cue story matched both a superficially dissimilar analogue and a superficially similar disanalogue with no structural overlap. They found a prevalence of structurally based over superficially based retrievals, supporting the dominance of structural similarities in analogical retrieval. A similar pattern of results was obtained by the same authors when investigating the retrieval of personal experiences elicited by target cues embodying a familiar category (e.g. *making up an excuse*) (Raynal et al., 2018, 2023, see also Blanchette & Dunbar, 2000).

Following on from Raynal et al.'s (2020) study, Trench et al. (2020) tested whether superficially similar disanalogues sharing a higher number of object similarities would prevail over superficially dissimilar analogues sharing familiar structures in retrieval. The superficially similar disanalogues shared either only objects (i.e. ‘O‐O matches’) or both objects and isolated relations (i.e. ‘R + O matches’). It was found that R + O matches, but not O‐O matches won the retrieval competition against superficially dissimilar analogues. In line, Minervino & Trench, 2024, (Experiment 2) obtained evidence that superficially similar disanalogues outnumber superficially dissimilar analogues sharing familiar structures while investigating the retrieval of public events, supporting surface dominance. However, another interpretation of the results of these two studies, which would be consistent with the structural superiority hypothesis, is that superficially similar disanalogues still share some kind of structural overlap. For instance, superficially similar disanalogues could both involve *someone who is supposed to spend money on reparations while she does not have much money* (R + O matches, Trench et al., 2020) or *authorities taking actions to help endangered species to recover their habitat* (Minervino & Trench, 2024). Overall, it appears that research has not reached a consensus on the respective roles of surface and structural similarities in retrieval and that further research is needed to clarify this empirical question.

Aside from the lack of agreement between the conclusions reached by traditional empirical studies, the validity of the most widely used, ‘two‐phase’, experimental paradigm itself has been the subject of serious criticisms (Day & Goldstone, 2012; Dunbar, 2001; Hofstadter & Sander, 2013; Minervino & Trench, 2024; Popov et al., 2017, Popov et al., 2020). In this experimental paradigm, one or several source situations (i.e. stories or problems) are presented to the participants before the introduction of target cue situation(s) sharing different possible kinds of similarities with the initial stimuli (Catrambone, 2002; Gentner et al., 1993; Gick & Holyoak, 1980; Holyoak & Koh, 1987; Kubricht et al., 2017; Raynal et al., 2020). However, it has been argued that naturalistic settings in which one produces analogies with one's own sources to attain personal goals foster a deeper encoding than the traditional task of reading narratives during an experiment (Dunbar, 2001; Hammond et al., 1991). In the current contribution, we point out that there is another crucial difference between traditional experimental settings and naturalistic ones, which relates to the format of the incoming situations. Whereas research on analogical retrieval has largely focused on written verbal stimuli, perceptual events in everyday life are typically dynamic, multimodal and crowded (Schmuckler, 2001; Sonkusare et al., 2019; Weinberger et al., 2022). How might findings on the role of surface and structural similarities in retrieval stemming from the presentation of events in written format generalize to their perceptual counterparts involving visual scenes and spoken language?

### The influence of events' perceptual properties on retrieval

Prior literature suggests that the perceptual nature of real‐life events may affect the determinants of analogical retrieval in several ways. First, there is evidence that presenting verbal experimental stimuli in spoken rather than written format increases the retrieval rate of superficially dissimilar analogues, while the retrieval frequency of superficially similar disanalogues remains unchanged (Markman et al., 2007). According to the authors, spoken discourse may be more conducive to encoding structures since it causes a lesser memory load than reading texts. In addition, people may be more practiced at extracting the main point from a spoken discourse rather than from a written text.

The visual properties of real‐life events may also affect the determinants of analogical retrieval compared to written texts. On the one hand, the visuospatial nature of events appears to promote structurally based retrievals. Pedone et al. (2001) demonstrated that presenting dynamic visuospatial displays with sparse surface details (e.g. arrows converging on a central target along different paths, to illustrate a military strategy) led to higher rates of spontaneous transfer than written texts or static visual descriptions (see also Day & Goldstone, 2011). This may be because such dynamic displays promote the encoding of the relevant spatial relations. On the other hand, real‐life events can generally create more detailed mental representations than written texts (Mazzoni et al., 2014). For instance, a scene of a man riding a horse contains many details about the horse and the rider that the verbal description ‘A man riding a horse’ does not necessarily convey. One possibility is that perceptually rich events exhibiting irrelevant surface details distract from structures and hinder analogical reasoning (Goldstone & Sakamoto, 2003; Markman & Gentner, 1993; Sloutsky et al., 2005; Thibaut et al., 2010). Thus, there are both advantages and disadvantages associated with the processing of perceptual events compared to written texts regarding the reliance on structural similarities during retrieval.

The role of surface similarities in analogical retrieval may also be influenced by the visual content of real‐life events. Interestingly, Mazzoni et al. (2014) showed that pictures (e.g. a picture of a *ball*) are less likely than written words (e.g. ‘ball’) to elicit the retrieval of autobiographical memories (e.g. a memory involving a *ball*). This may be because participants generate their own personally relevant details to represent written words, which provides additional retrieval cues for past experienced events (see also Mace, 2004). It may therefore be the case that surface similarities exert a weaker role in retrieval when they are visual rather than verbal.

Lastly, the fact that real‐life events are typically multimodal, involving both spoken discourse and visual content, may favour the implementation of structurally based retrievals. Kubricht et al. (2017) demonstrated that spoken problem descriptions accompanied by dynamic visuospatial displays allowed participants to process structural encodings, resulting in particularly high rates of structurally based retrievals. Grounding spoken verbal descriptions in dynamic perceptual experiences may provide the benefits associated with multimedia instruction. Indeed, Reed (2006) has reviewed evidence that mobilizing both verbal and visual codes increases recall, reduces interferences between co‐presented elements, allows for complementary information to be provided and promotes a deeper understanding of the situations. Thus, the current literature suggests that the processing of realistic perceptual events may promote the reliance on structural similarities to the extent that they involve a multimodal integration of spoken language and dynamic visual scenes. Furthermore, it might be the case that surface similarities between visual scenes are less effective than those expressed by verbal descriptions in eliciting superficially based retrievals. In contrast, perceptual events tend to induce more detailed mental representations than written texts, which may be less favourable for extracting abstract structures and using them as retrieval cues.

Following criticisms addressed to the two‐phase paradigm, researchers have investigated whether previous findings generalize to the retrieval of memories encoded during real‐life interactions, such as films, public events or autobiographical memories (Blanchette & Dunbar, 2000; Minervino & Trench, 2024; Olguín et al., 2022; Raynal et al., 2018, 2023; Trench & Minervino, 2015). In doing so, they have documented the retrieval of realistic perceptual events that are dynamic, multimodal and crowded. However, potential differences observed between the retrieval of real‐life memories and written texts may be imputable to factors other than the presentation format of the situations. For instance, it has previously been pointed out that retrieving real‐life events generally involves longer delays between the presentation of the source and the target cue, a greater number of irrelevant sources stored in LTM, but also encoding contexts that are more conducive to deep encoding than in the two‐phase paradigm (Chen et al., 2004; Dunbar, 2001; Gentner et al., 2009; Minervino & Trench, 2024).

Alternatively, the use of a two‐phase paradigm with stimuli characterized by a more ecological format than written texts may be a more appropriate approach to assess how previously observed patterns of analogical retrieval generalize to perceptual events. Namely, video clips of real‐life events have been widely used as experimental stimuli within the field of episodic memory retrieval (Baldassano et al., 2018; Congleton & Berntsen, 2020; Scheurich et al., 2021; Zacks, 2020). They are particularly valid experimental stimuli as they reproduce the dynamic, multimodal and crowded nature of everyday life experiences (Schmuckler, 2001; Sonkusare et al., 2019), while allowing researchers to precisely control the characteristics of the events presented to the participants (Congleton & Berntsen, 2018; Hermann et al., 2021; Saint‐Laurent et al., 2014). Of particular interest for the current study, Congleton et al. (2021) investigated how auditory and visual cues influence the retrieval of perceptual events. Specifically, they assessed whether the presentation of still images (e.g. a telephone) or sounds (e.g. a telephone ringing) from a previously presented video clip were more likely to elicit involuntary episodic retrievals. The main findings were that participants reported involuntary episodic retrievals when confronted with both types of cues, but that they did so more frequently in response to visual rather than auditory cues. This result comforted the idea that surface similarities in terms of sound and visual content exert a significant influence on the retrieval of perceptual events, with visual cues dominating over auditory ones (Berntsen, 2009). However, less is known about whether structural similarities can overcome such surface similarities in driving the retrieval of video clips describing real‐life perceptual events.

### Experimental overview

In the current study, we aim to inform participants' capacity to extract structures from perceptual events to retrieve superficially dissimilar analogues. More specifically, a major goal underlying the following experiments was to assess whether participants are able to bypass surface similarities between realistic perceptual events to retrieve cases based on structural similarities. To test this hypothesis, we built an experimental paradigm based on the traditional experimental design used to investigate analogical retrieval (Gentner et al., 1993; Raynal et al., 2020; Wharton et al., 1996), while borrowing some aspects from experiments documenting the role of perceptual similarities in episodic memory retrieval (Congleton et al., 2021; Congleton & Berntsen, 2020; Hermann et al., 2021). In line with typical story‐recall experiments, participants were presented with a set of source stories prior to the introduction of the target cue. Importantly, video clips were used instead of written narratives in order to more closely resemble the kind of perceptual experience that characterized the processing of real‐world events. They involved dynamic physical and verbal interactions between agents within realistic contexts typical of everyday life (e.g. *university*, *birthday party*, etc.). In order to pit surface similarities against structural similarities, the target cue shared only surface similarities with one source and only structural similarities with another source. Based on the idea that analogies are routinely implemented to make sense of incoming experiences (Chalmers et al., 1992; Hofstadter & Sander, 2013; Schank, 1999), our prediction was that structural similarities overcome surface similarities in the retrieval of realistic perceptual events. In other words, we predict that superficially dissimilar analogues would be retrieved more frequently than superficially similar disanalogues.

## EXPERIMENT 1A

### Method

#### Participants

One hundred and twenty‐nine French‐speaking participants volunteered to take part in the experiment. Recruitment took place in the library of the University of Paris VI (mean age = 21.9; *SD* = 2.5; 59 women) and the participants were undergraduate students from various disciplines. Participants received no compensation for their participation.

#### Materials

A competition paradigm was adopted, in which two types of matches preserving similarities with the target cue are both stored in LTM in order to mimic prevalent real‐life conditions in which superficially dissimilar analogues and superficially similar disanalogues compete for retrieval (Raynal et al., 2020; Trench et al., 2020; Wharton et al., 1994, 1996). Precisely, of the six source video clips, one was a superficially dissimilar analogue, one was a superficially similar disanalogue and the four remainders were superficially dissimilar disanalogues (i.e. distractors). The full set of stimuli and the data supporting the findings of this study are available here (Videos S1–S6).

**FIGURE 1:**
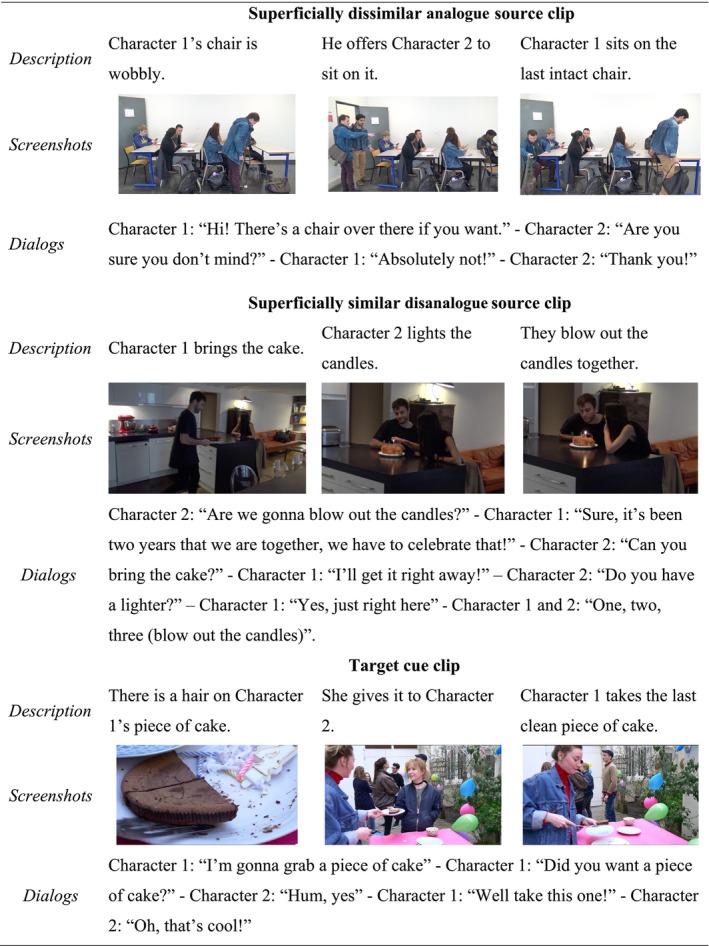
Description of the video clips with surface and structural similarities (set 1).

The video clips lasted about 30 s (*M* = 32.88; *SD* = 2.17) and described scenarios of everyday interactions with realistic, perceptually rich visual contexts (e.g. a movie set with party balloons, garlands, candies, plates, plants, etc., placed in a courtyard), as well as auditory information (e.g. spoken dialogues, see Figure 1). All actors were young adults and never appeared in more than one video clip that was presented to a given participant.

Critically, superficially dissimilar analogues were constructed to preserve only structural similarities, whereas superficially similar disanalogues were designed to share only surface similarities. Surface similarities were introduced in the form of a similar context (i.e. scenarios about *birthday celebrations*) and a similar salient object (i.e. the presence of a *chocolate/sponge cake with candles*). This object was the main focus of the characters' attention, and it was made even more salient by one of the actors explicitly labelling it (i.e. saying ‘cake’ to refer to the object). In this way, surface similarities were introduced at the level of both visual content and spoken language. The structural overlap involved novel structures (e.g. *offering a defective item to someone else to get the last good item for oneself*) referring to familiar categories (e.g. *politeness, benefits, perfidy*, etc.) (Raynal et al., 2020). For instance, one of the target cues described a guest at a birthday party who sees a hair on her piece of cake and subsequently gives it to another guest in order to take the last clean piece of cake on the tray (see Figure 1). In this set of clips, the superficially dissimilar analogue source described a student who, after noticing that his chair was wobbly, offered it to another student who arrived in the classroom and sat on the last intact chair available. The competing superficially similar disanalogue source showed a couple celebrating their 2 years relationship anniversary by blowing out candles on a cake.

In order to ensure that the proportion of structurally based retrievals obtained in this study is not tight to a specific case of structural similarities, two sets of six source video clips and one target cue video clip were constructed. The only difference between these two sets was the structure shared by the superficially dissimilar analogue source and the target cue. While the actors and contexts of the superficially dissimilar analogue source, as well as those of the target cue, were kept the same across the two sets, they involved the structure *offering a defective item to someone else to get the last good item for oneself* in the first set and the structure *giving up on the last item to offer it to someone else* in the second set. This manipulation allowed us to test whether retrieval can be based on different types of structure, while taking practical constraints into consideration (i.e. using the same movie set and actors in the two versions of the superficially dissimilar analogue sources and target cues). Notably, it would ensure that structurally based retrievals are not circumscribed to superficially dissimilar analogues in which agents have negative intentions (Shahbazyan et al., 2014). Half of the participants received the first set of video clips, and the other half received the second set.

#### Procedure

After providing an informed consent, participants were accompanied in individual boxes where they sat in front of a desk on which there was a laptop with audio headphones and an experimental booklet. Participants had to press the ‘start’ button when they were ready to begin the experiment to launch a sequence that introduced all the instructions and video clips. The order of presentation of the video clips within a sequence was semi‐randomized so that the superficially dissimilar analogue and superficially similar disanalogue sources never appeared as the first or the last video clip (an example of sequence presented to the participants is provided here). The sound level of the sequence was fixed before the beginning of each testing, and the participants were indicated that they could adjust it if needed. They were also informed that they were not allowed to pause, rewind or fast forward the sequence. An experimenter stood directly outside of the experimental box throughout the experiment in case the participant needed clarification.

Participants were asked to watch the video clips carefully so as to be able to remember them later. In line with the procedure adopted in previous story‐recall experiments to ensure a minimal depth of processing of the experimental stimuli (Catrambone, 2002; Raynal et al., 2020; Trench et al., 2020; Wharton et al., 1996), the participants were asked to rate each video clip on a 10‐point scale on two of four alternating dimensions (imageability, pleasantness, meaningfulness, interestingness). After the presentation of the six source video clips, a 5 min filler task (i.e. divergent thinking task) was presented on the screen and had to be completed in the booklet. Finally, the recall instruction preceded the presentation of the target cue video clip. Participants were told that they would be presented with a new video clip and that they would have to indicate whether it reminded them of a previously watched video clip. After the target cue was presented, participants were asked to report as many details as possible about the retrieved video clip, if they retrieved one, and to report only the video clip that best matches the target cue in case they recalled several video clips (Gentner et al., 1993; Raynal et al., 2020). At the end of the sequence, participants were asked for their personal information and were thanked for their participation. The entire experiment took approximately 15 min.

#### Sample size

A power analysis was performed in G*Power 3.1.9.7. (Faul et al., 2007) to approximate the sample size needed to detect significant differences between the retrieval of superficially dissimilar analogues and superficially similar disanalogues for each of the two sets of video clips. The number of responses required for each set was calculated based on an effect size of *φ* = 0.61 obtained by Olguín et al. (2022) when comparing the number of superficially similar/dissimilar analogues retrieved by participants from their own experience. Sixty responses per set were required for a power of >0.90 to detect an effect for the chi‐square analysis at an alpha level of .01. During the recruitment phase, the experimenters scheduled experiments with 140 participants to guarantee a final sample of at least 60 participants per set. A total of 129 students eventually took part in the scheduled experimental session.

### Results and discussion

Each response was coded by attributing a score of 1 to the type of source that was recalled (i.e. superficially dissimilar analogue, superficially similar disanalogue or superficially dissimilar disanalogue). The source video clip that was retrieved was identified on the basis of the participants' mention of a distinctive object (e.g. ‘a flawed chair’, ‘the last chair’, ‘birthday’, ‘a cake’) or action present in it (e.g. ‘fixing a chair’, ‘giving up on a seat’, ‘celebrating a relationship’). If the description could not lead to a clear identification of a type of source video clip (*N* = 3), or if the participant did not report any retrieval (*N* = 4), the response was coded as a non‐retrieval. The main analyses focus on participants who reported only one source video clip in line with the instructions (c.f. Gentner et al., 1993; Raynal et al., 2020).^1^ Nine multiple retrievals were identified in the present experiment (seven responses referring to both the superficially dissimilar analogue and the superficially similar disanalogue, one response reporting a superficially similar disanalogue and a superficially dissimilar disanalogue and one response referring to two superficially dissimilar disanalogues). There were no other criteria leading to the exclusion of any data.

**FIGURE 2:**
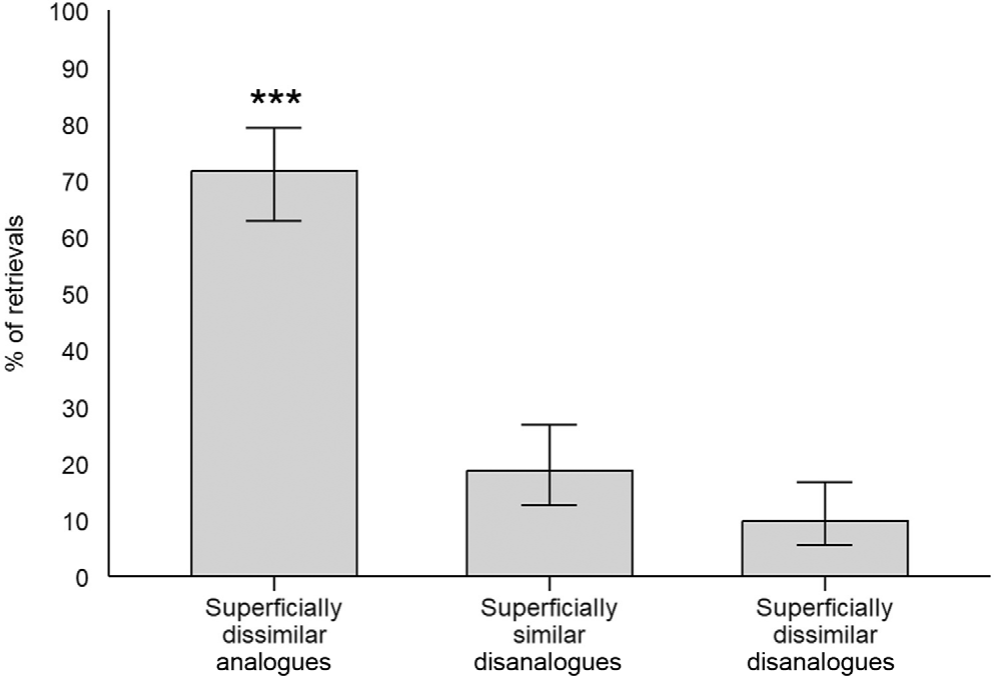
Proportion of each type of retrieval in Experiment 1A. Superficially dissimilar analogues, superficially similar disanalogues and superficially dissimilar disanalogues are videoclips that share, respectively, only structural similarities, only surface similarities, or neither kind of similarities. Error bars represent 95% confidence intervals. ***p < .001.

Among the 113 participants who retrieved only one source video clip, 71.68% retrieved the superficially dissimilar analogue, 18.58% retrieved the, superficially similar disanalogue and 9.74% retrieved a superficially dissimilar disanalogue (see Figure 2). A chi‐square test was performed to compare the number of participants retrieving either only a superficially dissimilar analogue or only a superficially similar disanalogue and revealed a significant difference (*χ* ^2^ (1, *N* = 101) = 35.29, *p* < .001, *φ* = .59).^2^ This difference was statistically significant both for both the group of participants who received the first (*χ* ^2^ (1, *N* = 55) = 30.56, *p* < .001, *φ* = .75) and the second (*χ* ^2^ (1, *N* = 47) = 7.68, *p* < .01, *φ* = .33) set of video clips.

The findings of Experiment 1A suggest that structural similarities have a more pronounced influence than surface similarities on the retrieval of perceptual events akin to those routinely encountered in real life. They extend previous work demonstrating the major role of structural similarities in analogical retrieval using written narratives as stimuli (Catrambone, 2002; Raynal et al., 2020; Wharton et al., 1996) to the retrieval of dynamic experiences exhibiting a complex array of perceptual features such as those typically encountered in real life.

Our interpretation of the results is that the dominance of superficially dissimilar analogues over superficially similar disanalogues originates from the greater influence of structural similarities over surface similarities in driving retrieval. However, an alternative explanation could be that the surface similarities that were introduced between the superficially similar disanalogues were not salient or numerous enough to drive retrieval (Trench et al., 2020). To control this possibility, we replicated the design of Experiment 1A while altering the superficially dissimilar analogues so as to remove their structural overlap with the target cue. If the surface similarities were strong enough to drive retrieval, but were outcompeted by the structural similarities, then superficially similar disanalogues should be retrieved more frequently than superficially dissimilar analogues when deprived of structural similarity.

## EXPERIMENT 1B

### Method

#### Participants

Fifty‐six undergraduate students took part in the experiment during a psychology course (mean age = 24.3; *SD* = 7.8; 34 women). The sample size needed to show a significant effect was approximated using a similar method as in Experiment 1A. As the target cue was the only element to vary between the two sets of stories, and since these two target cues had almost identical surface features, the data from the two sets were analysed together. Thus, we targeted a total sample size of 60 participants and achieved 56 participants for practical reasons related to the fact that the experiment was conducted during a class.

#### Materials

The two story sets were taken from Experiment 1A with the only modification being that the superficially dissimilar analogue was replaced by a new video clip. This video clip involved the same actors and movie set as the superficially dissimilar analogue source video clip from the previous experiment. However, the scenario was altered to suppress the structural overlap with the target cue. In this new superficially dissimilar source video clip deprived of structural similarities, Character 1 enters the classroom, says hello to Character 2 who is seated in the front row, and offers the latter to sit with him at the back of the classroom. At the end of the video clip, the two characters are sitting at the same table talking to each other. This video is 32.13 s long.

#### Procedure

The procedure of Experiment 1A was replicated. However, the participants completed the task in a classroom with individual computers and headphones. All participants received the same source video clips. Half of the participants received the target cue video clip from the first set, and the other half watched the target cue video clip from the second set.

### Results and discussion

Eight participants did not retrieve any source video clip and three of the responses could not lead to a clear identification of a source video clip. Three participants retrieved multiple source video clips. As expected, among the 42 participants who retrieved a single source, most retrieved the superficially similar disanalogue source video clip (88.10%), and only a few retrieved the superficially dissimilar source deprived of structural similarities (7.14%) or the (other) superficially dissimilar disanalogues (4.76%). The difference between the number of superficially similar disanalogues and superficially dissimilar sources deprived of structural similarities that were retrieved was significant (*χ* ^2^ (1, *N* = 39) = 28.9, *p* < .001, *φ* = .86).

Experiment 1B shows that the surface similarities introduced in Experiment 1A were strong enough to induce a near‐perfect retrieval rate in the absence of a competing structural match. The data confirm that the reason why superficially dissimilar analogues were retrieved more frequently than superficially similar disanalogues in Experiment 1A is that structural similarities prevail over surface similarities in guiding memory access.

Our interpretation of the results from Experiment 1A is that the structural cues contained in the target cues were particularly efficient in eliciting the retrieval of the superficially dissimilar analogues stored in memory. However, it could be argued that the results do not completely rule out the possibility that the retrieval mechanism is mainly based on surface similarities. In this view, the relatively small number of source video clips would have allowed participants to use a ‘reactivation‐mapping’ strategy, serially comparing the target cue with each of the six previously watched sources in order to find the structural match. The implementation of this cognitively demanding strategy seems very unlikely, as participants would have perfectly fulfilled the requirements of the recall task by simply reporting the retrieval of the superficially similar disanalogue. Nevertheless, the fact that the use of this strategy would lead to a similar pattern of results to those obtained in Experiment 1A invited us to control for this possibility in Experiment 2. In this experiment, we increased the number of source video clips to make even less likely the possibility that participants would use a reactivation‐mapping strategy.

## EXPERIMENT 2

### Method

#### Participants

One hundred and twenty students (mean age = 23.7; *SD* = 6.3; 63 women) volunteered to participate in the experiment in the library of the University of Paris VI. We used the same method as in Experiment 1A to approximate the sample size needed to show a significant effect in Experiment 2.

#### Materials & procedure

The materials and procedure were replicated from Experiment 1A, except that the number of source video clips was increased. As the traditional story‐recall paradigm from which the present video clip‐recall design was inspired may induce a shallow processing of the experimental stimuli (Blanchette & Dunbar, 2000), presenting an excessively high number of source stories could discourage participants from engaging in a deep understanding of the video clips (Raynal et al., 2020). In order to make the use of the reactivation‐mapping even less likely than in the previous experiment, but without discouraging participants to pay attention to each video clip, three additional superficially dissimilar disanalogues were introduced within the sources from Experiment 1A. Hence, participants watched nine source video clips (one superficially dissimilar analogue, one superficially similar disanalogue and seven superficially dissimilar disanalogues) and one target cue video clip (mean duration = 31.83 s; *SD* = 3.58 s).

### Results and discussion

One participant did not retrieve any source video clip and another participant's response could not lead to a clear identification of any source video clip. Among the 118 participants who retrieved at least one source video clip, 10 were excluded from the main analyses because they retrieved several sources (seven participants retrieved both the superficially dissimilar analogue and the superficially similar disanalogue, two participants retrieved the superficially similar disanalogue and a superficially dissimilar disanalogue, and one retrieved two superficially dissimilar disanalogues).

**FIGURE 3:**
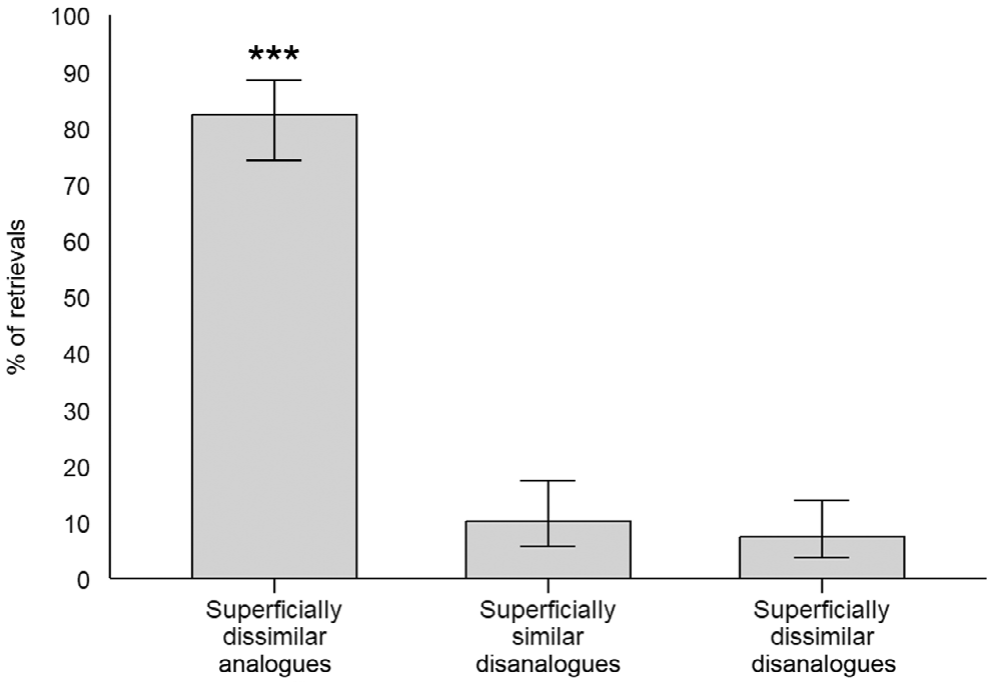
Proportion of each type of retrieval in Experiment 1A. Superficially dissimilar analogues, superficially similar disanalogues and superficially dissimilar disanalogues are videoclips that share, respectively, only structural similarities, only surface similarities, or neither kind of similarities. Error bars represent 95% confidence intervals. ***p < .001.

In line with the results of Experiment 1A, participants retrieved more superficially dissimilar analogues (82.41%) than superficially similar disanalogues (10.19%) and superficially dissimilar disanalogues (7.41%, see Figure 3). Again, a chi‐square test revealed that the difference between the number of superficially dissimilar analogues and superficially similar disanalogues that were retrieved was significant (*χ* ^*2*^ (1, *N* = 99) = 60.83, *p* < .001, *φ* = .78).^3^ This was the case for both the group of participants who received the first set of video clips (*χ* ^*2*^ (1, *N* = 55) = 44.64, *p* < .001, *φ* = .90) and the second set of video clips (*χ* ^*2*^ (1, *N* = 43) = 17.82, *p* < .001, *φ* = .64).

The findings from Experiment 2 converge with those of Experiment 1A and provide further evidence for the structural superiority in the retrieval of perceptual events. Because it is extremely unlikely that participants would have reactivated the nine source video clips to engage in a mapping of each of them to the target cue video clip, the predominance of superficially dissimilar analogues over superficially similar disanalogues in the current experiments can be more safely attributed to a structurally based retrieval process. Moreover, if the pattern of results of Experiment 1A were due to participants' use of a reactivation‐mapping strategy from the participants, one would have expected an impairment in the rate of structurally based retrievals in Experiment 2 associated with the greater cognitive demands of reactivating and mapping a greater number of source video clips to the target cue video clip. However, the retrieval rate of superficially dissimilar analogues was not negatively affected by the introduction of a greater number of irrelevant source video clips. Thus, participants seem to focus on structural similarities when retrieving perceptual events.

## GENERAL DISCUSSION

The aim of this paper was to assess the determinants of analogical retrieval in conditions where the experimental stimuli more closely reflect the dynamic, multimodality and perceptual crowdedness of real‐life experiences than in previous experiments. A new experimental paradigm was designed in which participants were presented with video clips depicting physical and verbal interactions between agents in real‐life contexts. In the spirit of classical story‐recall tasks, we introduced either surface or structural similarities between the video clips so as to assess which type of similarities prevails in driving the retrieval of realistic stimuli. In Experiment 1A, participants retrieved superficially dissimilar analogues more frequently than superficially similar disanalogues, demonstrating that structural similarities outcompete surface similarities in guiding retrieval. In Experiment 1B, we showed that superficially similar disanalogues became predominantly retrieved when the structural overlap with the competing superficially dissimilar analogue was neutralized. This finding shows that the surface similarities that we introduced in our experimental stimuli were sufficient to elicit near‐ceiling retrieval rates, thus confirming that the dominance of superficially dissimilar analogues in Experiment 1A was due to structural similarities overcoming surface similarities in analogical retrieval. Lastly, Experiment 2 demonstrated that structurally based retrievals remain prevalent when a greater number of sources were stored in LTM, making the implementation of the reactivation‐mapping strategy extremely unlikely. Taken together, the results from the current study show that participants are able to extract structures from realistic perceptual events in order to use them as preferential retrieval cues.

Prior experimental research has yielded inconsistent results regarding the determinants of analogical retrieval. While some studies found that structural similarities play a minor role and are overshadowed by surface similarities during retrieval (Gentner et al., 1993; Jamrozik & Gentner, 2020; Minervino & Trench, 2024), others obtained an opposite pattern of results supporting the structural superiority (Blanchette & Dunbar, 2000; Raynal et al., 2018, 2020, 2023). In line with several authors, we have argued that one reason for the difficulty to retrieve superficially dissimilar analogues in several previous studies (e.g. Gentner et al., 2009; Gick & Holyoak, 1980; Jamrozik & Gentner, 2020; Minervino & Trench, 2024, Experiment 1; Ross, 1987; Snoddy & Kurtz, 2021), is not related to the nature of the retrieval mechanism per se, but rather to the difficulty of encoding particularly unfamiliar structures (Dunbar, 2001; Popov et al., 2017; Raynal, 2022; Sander & Richard, 2005). In this context, only experts who have acquired the relevant knowledge are able to encode abstract structures and to use them as retrieval cues (Goldwater et al., 2021; Kretz & Krawczyk, 2014; Novick, 1988). However, our results show that individuals without specific expertise are also able to base retrieval on structural similarities as long as the situations can be interpreted through sufficiently familiar categories (e.g. *politeness*, *benefits* and *perfidy*). Interestingly, other studies have found evidence supporting the surface superiority when investigating the retrieval of events exhibiting familiar structures (Gentner et al., 1993; Minervino & Trench, 2024, Experiment 2; Trench et al., 2020). Our interpretation of these findings is that residual structural similarities between superficially similar situations have inflated their retrieval rates. In line, participants have previously been shown to preferentially retrieve memories sharing both surface and familiar structures (Olguín et al., 2022; Raynal et al., 2023; Trench & Minervino, 2015). We suggest that neutralizing any structural overlap between the superficially similar disanalogues (involving friends at a birthday party versus a couple celebrating their relationship anniversary) was a critical experimental requirement for revealing the structural superiority in the current study.

Another implication of the present study is a mutual enrichment of the closely related fields of analogical retrieval and episodic memory retrieval. Establishing tighter connections between the two literatures is highly desirable as they share a common interest in the type of cues eliciting the retrieval of past events from memory (Berntsen, 2007; Gentner & Maravilla, 2018; Holyoak, 2012; Mace, 2004). By using video clips as proxies for real‐life events in the spirit of research on episodic memory retrieval, we were able to investigate analogical retrieval in more ecological settings. Specifically, our results highlight the robustness of structurally based retrieval by showing that structural cues prevail even when dealing with realistic events exhibiting a complex array of perceptual data. Reciprocally, investigating the role of structural similarities in retrieving video clips from memory has also been insightful for research on episodic memory retrieval. While this literature has extensively studied the effect of perceptual cues (Berntsen, 2009; Congleton et al., 2021), our data suggest that they are not the only factor influencing retrieval, as structural cues were shown to be prevalent over visual and auditory cues in our experiments.

A wealth of research has demonstrated that comparing superficially dissimilar analogues is an efficient way to build novel abstract relational categories organized around common structures (e.g. *robbery*, *politeness* and *procrastination*) (Catrambone & Holyoak, 1989; Gentner et al., 2011; Goldwater & Gentner, 2015; Kurtz & Gentner, 2001). The surface superiority account of analogical retrieval suggests that spontaneous comparisons are problematic as a route to experiential learning, as the retrieval system would mainly lead to the retrieval of past events sharing surface rather than structural similarities with incoming situations. Nevertheless, it has been argued that several factors compensate for the limitations of the retrieval system (Gentner & Medina, 1998). First, the *kind world* hypothesis suggests that most things that look alike are also relationally similar, so that superficially based retrievals often lead to the retrieval of structurally sound matches. In addition, irrelevant past events that come to mind can be easily rejected during the subsequent mapping process. Another point relates to the role of language, as similar labels invite comparisons that could have gone unnoticed. The findings from the current research provide an even more optimistic view of spontaneous comparisons as a route to experiential learning. The fact that perceptual events that share novel structures (e.g. *offering a defective item to someone else to get the last good item for oneself*) are efficiently retrieved from memory suggests that retrieval allows adults to make fruitful spontaneous comparisons to build new abstract relational categories. However, we have argued that a condition for structurally based retrieval to occur is that the structures refer to familiar categories (e.g. *politeness, benefits, perfidy*). This raises the question of the developmental trajectory of analogical retrieval, as children may not initially be sufficiently familiar with the critical abstract relational categories to process structurally based retrievals. One possibility, which would require further empirical investigation, is that young children and adult learners initially tend to retrieve superficially similar analogues, and progressively grasp their structural commonalities in a way that allows for more structure‐oriented retrievals (Gentner, 2010; Hofstadter & Sander, 2013).

Grounded theories of cognition have put forward that the representation of abstract concepts is supported by perceptual processes such as mental imagery (Barsalou, 1999; Day & Goldstone, 2012; Lakoff & Johnson, 1999). In line, some authors have obtained evidence that the representation of abstract concepts is supported by the perceptual simulation of concrete aspects of their instances (e.g. contextually related entities, Wiemer‐Hastings & Xu, 2005). Using a lexical decision task, McRae et al. (2018) found that pictures of real‐world situations and abstract concepts (e.g. *share* and *anger*) prime one another, which was interpreted as showing that situation‐based perceptual knowledge underlies such concepts. The data from our experiments support the existence of a close link between perceptual and abstraction processes in that they illustrate that perceptual events akin to those encountered in the real world tend to activate abstract concepts (McRae et al., 2018). Furthermore, Day and Goldstone (2011) have provided an explanation for their results that may also be compatible with those of current study. They have suggested that the spontaneous transfer that they observed between superficially different animated problem descriptions may have been mediated by the extraction of a common mental model that integrates both perceptual (e.g. spatial) and meaningful conceptual content. In this view, the structurally based retrievals observed in our experiments may have resulted from the extraction of perceptually grounded representations (e.g. *giving a piece of cake with a hair on it*, or *giving a flawed chair*) that supported the extraction of abstract meanings (e.g. *offering a defective item*). The findings from Experiment 1B, which showed that the superficially dissimilar analogues deprived of structural similarities were no longer retrieved, even though they involved very similar spatial configurations, confirm that abstract meanings were crucial in eliciting structurally based retrievals.

Relatedly, our findings can be addressed in the light of the literature documenting the advantages and disadvantages of using concrete versus abstract materials to promote analogical retrieval and subsequent transfer (Day & Goldstone, 2011; Fyfe et al., 2014; Kaminski et al., 2009; Sloutsky et al., 2005). This research has provided evidence that a key drawback associated with the use of realistic examples is that they typically involve extraneous perceptual details that may distract from the relevant abstract principle that they instantiate. It follows that presenting idealized perceptual representations, which are sparser in surface details than more realistic ones, is an effective way to promote spontaneous transfer (Goldstone & Sakamoto, 2003; Kubricht et al., 2017; Pedone et al., 2001; Trench et al., 2023). However, the extent to which perceptual details impair transfer may depend on participants' familiarity with the abstract principle under scrutiny. This interpretation is supported by the fact that, in the current study, participants who had sufficient abstract knowledge about the stimuli did not appear to be distracted by the perceptual details displayed in the video clips and were able to implement structurally based retrievals. A better understanding of the circumstances under which perceptual details interfere with transfer could be achieved with future research investigating this potential interaction with participants' knowledge.

We might also consider potential limitations of the current study and the need for further investigation. One potential limitation is that our findings are based on a set of stimuli that included only one superficially dissimilar analogue (with two alternative structures), one superficially similar disanalogue and one target cue (also with two alternative structures). The decision not to include more of these critical video clips in the set presented to each participant was motivated by the prior consideration that presenting a great number of stimuli in the context of a two‐phase paradigm tends to induce a surface‐level encoding of the stimuli (Dunbar, 2001; Hammond et al., 1991; Hofstadter & Sander, 2013; Raynal et al., 2020). However, it would be useful to replicate the superiority of structural similarities with more variation in the set of stimuli containing the different types of similarities. Moreover, while the aim of our study was to assess how previous findings on analogical retrieval generalize to the retrieval of realistic events which are dynamic, multimodal and perceptually crowded, it does not allow us to determine which of these variables promote or hinder the reliance on surface or structural similarities in retrieval. It would be instructive to directly test how each of these factors influences the retrieval of perceptual events compared to written texts. Lastly, research testing the effect of easily understandable structures on the retrieval of past events has focused either on structures matching a preformed category (e.g. *sour grapes*, *making up an excuse*, *making a deal to avoid a bad situation*) or on structures whose encoding requires the combination of several preformed categories (e.g. *politeness, benefits, perfidy* in the current study). We believe that an insightful line of research would be to assess whether the retrieval of superficially dissimilar analogues is more likely to occur when the critical structure corresponds to a single preformed category in memory, or whether novel structures can be encoded as readily when they require the combination of multiple familiar categories (Olguín et al., 2022). More generally, a promising avenue for future studies may be to investigate how everyday life knowledge constrains encoding and retrieval.

## AUTHOR CONTRIBUTIONS

**Lucas Raynal:** Conceptualization; investigation; writing – original draft; methodology; data curation. **Evelyne Clément:** Conceptualization; methodology; writing – review and editing; supervision. **Emmanuel Sander:** Conceptualization; methodology; writing – review and editing; supervision.

## CONFLICT OF INTEREST STATEMENT

We have no conflict of interest to disclose. The original data and study analysis code will be made available on the corresponding author's OSF account prior to publication of this manuscript.

## Supporting information


Videos S1–S6.

## Data Availability

The data that support the findings of this study are openly available in OSF at https://osf.io/phnb2/?view_only=f20002eaa2384beabe44ee006a606f0d

## Footnotes

^1^
Complementary analyses were also performed on the number of each type of sources recalled while including participants reporting several retrievals.

^2^
An Additional McNemar test was performed on the data including participants who retrieved several source video clips and consistently showed that there was a significant difference between the number of participants retrieving the superficially dissimilar analog and the number of participants retrieving the superficially similar disanalogs (*χ* ^2^ (1, *N* = 121) = 32.66, *p* < .001, *OR* = 3.68).

^3^
An Additional McNemar test performed on the data including participants retrieving several types of sources converged with the main analyses and revealed a significant difference between the number of superficially dissimilar analogs and the number of superficially similar disanalogs that were retrieved (*χ* ^2^ (1, *N* = 117) = 55.15, *p* < .001, *OR* = 6.85).
